# Supplementary material for: Risk stratification of atrial fibrillation and stroke using single nucleotide polymorphism and circulating biomarkers
Source: PLoS One. 2023 Oct 12;18(10):e0292118. doi: 10.1371/journal.pone.0292118 (PMC10569505; doi:10.1371/journal.pone.0292118)
Supplement: Attachment — Submitted filename: Response to revieweres.docx [file pone.0292118.s004.docx]

Please use the space provided to explain your answers to the questions above. You may also include additional comments for the author, including concerns about dual publication, research ethics, or publication ethics. (Please upload your review as an attachment if it exceeds 20,000 characters)

*Reviewer #1: This single-center study prospectively collected 600 blood samples from the refereed patients and healthy volunteers. The authors compared the presence of SNPs that have already been shown to contribute to the development of AF between participants with and without a history of paroxysmal AF and examined their diagnostic ability for both paroxysmal AF and stroke in this population by ROC curves. The concept is interesting; however, the reviewer is concerned that the framework and methodology are not carefully described. Specific comments are as follows.

The most significant limitation of this study is that the authors collected blood samples from participants already diagnosed with AF or stroke and identified them. The relationship is unclear whether the morbidity caused the SNP mutation or vice versa. It is most desirable to predict future events from a disease-free state. Since several clinical risk scores (CHADS, CHA2DS-Vasc) are used in routine clinical practice to predict stroke, showing additive values of this genetic risk score is valuable.*

We appreciate the reviewers’ valuable comments.

As the reviewer pointed out, the design of this study is a cross-sectional study comparing patients with AF with controls. Regarding SNPs, the genetic polymorphisms are determined at birth and do not change, thus we can exclude the possibility that AF cause SNP mutations. In terms of miRNAs and cell-free DNA, it is not clear to say the increased level of biomarkers is the cause or result of AF or strokes. We totally agree with the reviewer that a large-scale prospective study is required to answer this issue. This study is positioned as the preliminary study for coming large-scale prospective study. We added this point in the revised manuscript.

In addition, according to the reviewer’s suggestion, we calculated the risk score for stroke in PAF group, by combining CHADS_2_ score, genetic risk score, and cfDNA score. We added these results as Fig 4 in revised manuscript.

*This paper has two aims, to examine the validity of known SNPs associated with the development of AF and to investigate the relationship between these SNPs and a history of stroke. The Introduction needs to state why this study should be done clearly.*

Thank you for the comments regarding critical point. Our working hypothesis is the combined analysis including genetic information and circulating biomarkers contributed to increase the accuracy of the prediction of AF and stroke. And this cross-sectional study might work to choose promising items for prediction of AF or stroke. We added the sentence for the aim of this study.

*Various SNPs related to AF have already been reported. (Nat Genet. 2017 Jul 27;49(8):1286.) Still, the rationale for investigating these nine SNPs in this study must be clarified.*

As aforementioned, the main purpose of this study is to investigate if the integrated analysis combining SNPs and biomarkers improve the prediction accuracy for AF and strokes. Thus we chose SNPs which were already known in association with AF in Japanese population. It is out of our scope to find novel SNP in relation to the AF. We stated this point in revised manuscript.

*Definitions of critical variables should be described. It is unclear whether hemorrhagic infarction is included, the definition of paroxysmal, and the basis for the diagnosis.*

Thank you for raising important points. We included hemorrhagic infarction in stroke group but excluded cerebral and subarachnoid hemorrhage. We added that with the definition of strokes and paroxysmal AF, and the diagnostic basis for them.

*How were these 600 participants recruited? If the authors were initially designed to compare 300 participants, each paroxysmal AF vs. non-AF, that should be stated.*

As we stated previously, this study is positioned as preliminary study to pick up the measurement items for coming large-scale prospective analysis. Thus we picked up each 300 participants for PAF and control groups. We added this description.

*Reviewer #2: The authors aimed to evaluate the risk of paroxysmal AF (PAF) and stroke using genetic analysis and circulating biomarkers. They enrolled 600 adult subjects (300 PAF and 300 controls), and identified the genetic variation and biomarkers. The results showed genotyping identified 4 SNPs being associated with AF (rs6817105, rs3807989, rs10824026, and rs2106261). Circulating miRNAs and cfDNA did not show significant differences between PAF and control groups, but the concentration of cfDNA was higher in patients with a history of stroke. The authors concluded that the risk of PAF could be assessed by genetic risk score, and the risk of stroke may be evaluated by plasma cfDNA level. Although the data are interesting, there are several concerns.

First, the study consisted of a small number of PAF and control subjects. As indicated by the authors, early detection of PAF seems to be useful for early intervention that prevents progression of PAF to persistent AF. From the present small sample study, only a preliminary data may be obtained. How do the authors confirm that control group did not include PAF? Considering the similar clinical characteristics between the 2 groups, it may be difficult to exclude the possibility that control group includes some asymptomatic PAF patients.*

Thank you for valuable comments. As the reviewer pointed out, the large limitation of this study is its relatively small sample size. We described this point in limitation.

We defined the control group as having no record of AF in ECG, no record of irregular pulse by physical examination in the annual health check, and no symptoms suggesting AF. However, as the reviewer indicated, we cannot exclude the possibility that the control group might have asymptomatic AF episodes. We added the definition of control group in the Methods, and the description of limitation.

*Second, 4 SNPs they identified are already said to be associated with AF. What are the new findings shown in this study? Please clarify this.*

Thank you for the comments regarding critical point. As the reviewer indicated, all 4 SNPs are already known to be associated with AF in previous studies, and we have no new findings focusing only for SNPs. Our working hypothesis is the combined analysis including genetic information and circulating biomarkers contributed to increase the accuracy of the prediction of AF or stroke. And this cross-sectional study works to choose possible items for prediction of AF. We added the sentence for the aim of this study.

*Third, they found that the concentration of cfDNA was higher in patients with a history of stroke. Was this specific for PAF group? Which type of stroke was associated with the increased cfDNA level? Was it a cardioembolic stroke? Was the increase a cause or result? Please clarify this.*

Thank you for raising up the critical point. The level of cfDNA was similarly higher in the stroke group both in the PAF group and control group. And we extracted the information of the history of stroke from medical record. Thus these results indicated that cfDNA was the biomarker for stroke including all subtypes, but not a specific biomarker for cardiogenic stroke.

And we appreciate to give us another important point. Since this is the cross-sectional study, we cannot say the increased level of cfDNA is a cause or result. We need to clarify this with prospective study. We added the data this in the discussion in revised manuscript.

*Finally, this reviewer understands that this is a preliminary study for the large-size, prospective study in which the relation of genetic variation found in the* *study will be tested in a large number of subjects. If so, please state clearly in the manuscript.*

We really appreciate the reviewer’s important suggestion. The understanding of reviewer is totally correct. This paper is the preliminary study to choose items for coming large-scale study. We added the description in the revised manuscript.
