# Supplementary material for: Risk stratification of atrial fibrillation and stroke using single nucleotide polymorphism and circulating biomarkers
Source: PLoS One. 2023 Oct 12;18(10):e0292118. doi: 10.1371/journal.pone.0292118 (PMC10569505; doi:10.1371/journal.pone.0292118)
Supplement: Attachment — Submitted filename: 20230817_Response to revieweres_2.docx [file pone.0292118.s005.docx]

We appreciate the reviewers for taking time to review our manuscript and giving valuable comments.

Reviewer #1: The authors correctly addressed the comments, and the manuscript has been improved. However, the reviewer still wants to mention a few points.

The authors often used the phrase "prediction of AF or stroke," but this is misleading and only looks at the association with AF or stroke, given the nature of a retrospective cross-sectional study. Additionally, from the results of this paper, the authors should not conclude that "the risk of AF could be assessed by combining genetic risk scores." The authors should correct this throughout the paper.

Thank you for raising the point. We understand that the phrase "prediction of AF or stroke" is inadequate for this retrospective cross-sectional study. According to the reviewer’s suggestion, we changed that phrase to “estimate the association with AF or stroke” or “estimate　the AF or stroke group” throughout the manuscript. We also removed the phrase "the risk of AF could be assessed by combining genetic risk scores" in conclusion, and changed the entire paragraph. We remained using “predict/prediction” in the context of our final goal and the description of reference.

Detailed information, the calculation for genetic risk score, and the combined risk score for stroke by CHADS2 and genetic risk score (some readers will not understand what CHADS2 stands for) should be included in the Method section rather than the Results.

Thank you for the valuable comment. According to the reviewer’s comment, we added the definition and calculation formula of CHADS_2_ score and genetic risk score in Method section. We added the reference to explain CHADS_2_ score in revised manuscript.

Reviewer #2: Thank you for revising the manuscript according to the comments of this reviewer. Although there are limitations in this study, it would be useful for further investigation in a large number of patients. I do not have any other comments to this manuscript.

We really appreciate the reviewer’s valuable suggestions and comments. Thanks to the reviewer, we consider that our manuscript improved for taking time to review our manuscript and giving valuable comments.
